# Stakeholders’ perceptions of 10 years of the Global Action Plan for Influenza Vaccines (GAP) – Results from a survey

**DOI:** 10.1016/j.vaccine.2016.08.040

**Published:** 2016-10-26

**Authors:** Claudia Nannei, Shoshanna Goldin, Guido Torelli, Hiba Fatima, Kaveri Kumar, Oliver Bubb-Humfryes, Bo Stenson, Erin Sparrow

**Affiliations:** a World Health Organization, Switzerland; b Cambridge Economic Policy Associates (CEPA), United Kingdom

**Keywords:** Influenza vaccine, Survey, Seasonal influenza, Pandemic preparedness

## Abstract

Ten years after the launch of the Global Action Plan for Influenza Vaccines (GAP), the World Health Organization (WHO) surveyed stakeholders to understand their perceptions of what the programme had achieved. This article provides a summary of the findings; the full report will be available on-line on the GAP website in November 2016 (http://www.who.int/influenza_vaccines_plan/en/). Seventy-seven responses were received from stakeholders including medical doctors, national influenza center officials, country immunization programme teams, surveillance and disease centers, policy-makers, researchers, vaccine manufacturers, and non-governmental organizations from 28 countries, representing all six WHO regions.

Respondents cited GAP’s biggest successes as capacity building in developing countries; raising international awareness of global needs in the event of a pandemic; and collaborative alignment of influenza stakeholders. The most commonly reported challenges were the limited progress in development of a broadly protective or universal vaccine and the perceived absence of a major increase in seasonal demand. These findings aligned with the perception that less global progress had been made under the third GAP objective, focused on research and development of better vaccines, than on increasing seasonal vaccine use (objective 1) and pandemic vaccine production capacity (objective 2). Respondents explained what they saw as the major challenges to development of better vaccines, including to development of a universal influenza vaccine. The majority of respondents agreed that the goal chosen at the GAP II consultation is still relevant. Results highlighted the importance of promoting research and development of better vaccines, both for facilitating uptake of seasonal vaccines and for ensuring timely vaccine availability in the event of a pandemic. As the GAP concludes its mandate this year, these findings will contribute to discussions on the impact of programme closure and how to address the key issues facing influenza stakeholders thereafter.

## 1 Introduction

Following the influenza A(H5N1) outbreak in 2005, there was growing recognition that in the event of an influenza pandemic there would be a global shortage of vaccines. In addition, production capacity was concentrated in a few high income countries. Resource-constrained countries would face delayed, limited and possibly no access to vaccines. To address this, the World Health Organization (WHO) convened a consultation in 2006 which endorsed the Global Action Plan for Influenza Vaccines (GAP) [1].

The GAP provided a comprehensive strategy to address global scarcity and inequitable access to influenza vaccines in the event of a pandemic, with three objectives [1]:

Objective 1: Increase evidence-based seasonal vaccine use.
Objective 2: Increase global pandemic vaccine production capacity and strengthening national regulatory competencies.
Objective 3: Foster development of new influenza vaccines that are not only higher-yielding and faster to produce, but also inducing broader and longer protection.

The strategy was reviewed and updated at a global consultation in 2011 [2].

As WHO assesses the GAP’s impact over the last decade, this article offers insights into how government representatives, industry leaders, researchers and public health professionals perceive its successes and failures, as well as future actions that WHO and the global community should consider during the discussions at the final GAP consultation in November 2016. Stakeholder perceptions, from both the public and private sectors, are important as they all have key roles to play within the GAP framework [3]. Manufacturers who received technology transfer and grants can provide insightful input into the extent to which the GAP facilitated the establishment of influenza vaccine production. Public health officials can comment on how WHO coordinated this intersectoral approach with national authorities and on the needs and challenges of supporting policy implementation for influenza vaccination. Researchers can offer helpful perspectives on the R&D landscape and associated challenges. Input from these stakeholders provides a more holistic and accurate picture of the progress made by the GAP.

## 2 Methodology

WHO conducted an online survey between 16 December 2015 and 29 February 2016 [4]. Questions covered the following areas: the relevance of the immunization goal agreed upon at the GAP II consultation; the progress made with regards to the three GAP objectives; the quality of data on diseases burden and vaccine effectiveness; the factors influencing sustainable seasonal influenza vaccine manufacturing and uptake; pandemic preparedness, and progress in R&D. The survey was announced at several influenza-related meetings and through direct mailings to the participants of the first (2006) and second (2011) GAP consultations [5], as well as to umbrella associations such as the International Federation of Pharmaceutical Manufacturers and Associations (IFPMA) and the Developing Countries Vaccine Manufacturing Network (DCVMN), and to members of the WHO Strategic Advisory Group of Experts on Immunization (SAGE). The survey was also posted on the WHO and International Society for Influenza and Other Respiratory Virus Diseases (ISIRV) websites and advertised in several newsletters.

## 3 Survey questionnaire structure

The survey comprised a total of 22 questions including questions with yes/no answers, multiple-choice questions and open-ended questions. To understand views on progress made under the three GAP objectives multiple choice questions used a Likert scale with the following ratings: very good, good, poor, and very poor. Another type of multiple choice question was used to understand perception of the importance of specific issues, where numerical values were assigned for descriptive purposes as follows: very important, somewhat important, not important. This scale was used to measure stakeholder perception of the importance of pandemic influenza vaccine availability and sustainable influenza vaccine production. Unlike the Likert scale described above, this measures the degree of importance for each factor, rather than ratings above or below a neutral option. Although the results may have been influenced by the choice of scale, they are suitable for this survey which seeks to gauge the perceived *relative* importance of each factor, rather than the absolute importance of any single factor.

### 3.1 Analysis

Multiple-choice responses were converted into average scores to increase comparability. For example, if a question asked respondents to choose whether a certain factor was “very important”, “somewhat important” or “not important”, answers were given scores of 1, 0 and −1 respectively and averaged across the group. The resulting average number was used to compare relative importance of a certain factor. Responses were also calculated and analyzed for sub-groups based on respondent characteristics such as country of origin or field of work. Written responses were analyzed by being read in detail to identify common themes, informative points, and areas of strong opinion. We report here on a diverse array of responses to ensure that several different points of view are represented.

## 4 Participants

Seventy-seven responses were received from respondents in 28 countries. Respondents did not answer every question; the least answered question received 29 responses.

Fifty-eight percent of respondents represented the vaccine supply side: vaccine/pharmaceutical manufacturing (26%), vaccine research (22%) and biotechnology (10%). The remaining respondents were medical doctors, national influenza center officials and other staff from surveillance and disease centers, national policy/planning departments, WHO collaborating centers, multilateral organizations, country expanded programmes on immunization (EPI) and non-governmental organizations. Of the fourteen manufacturers that received GAP funding to initiate a technology transfer project, eleven participated in this survey.

**Fig. 1:**
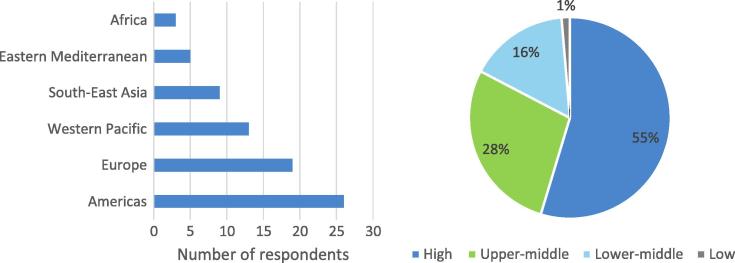
Geographical distribution of respondents.

Fifty-five percent of respondents were from high income countries, 28 percent from upper middle income countries, 16 percent from lower middle income countries, and 1 percent from a low income country (Fig. 1). All six WHO regions were represented, with 34 percent from the Americas, 25 percent from Europe, 17 percent from the Western Pacific, 11 percent from South East Asia, 6 percent from the Eastern Mediterranean, and 4 percent from the Africa region (Fig. 1). The remaining 3 percent identified themselves as working for global organizations.

## 5 Key findings

This article presents an overview of a selection of the survey questions and responses, as the complete analysis will be posted on the WHO website before the consultation planned for November 2016 to mark the end of the ten years of the GAP [6]. The answers presented in this article were chosen to provide a representation of the views of a variety of the respondents (medical doctors, manufacturers, national influenza center officials, policy makers, NGOs, etc.).

### 5.1 Relevance of GAP goal

The GAP II consultation, held in 2011, agreed that in order to bring pandemic virus transmission under control, 70% of the global population should be immunized with two doses of vaccine within six months of the pandemic candidate vaccine virus being available, given the effects of herd immunity.

**Fig. 2:**
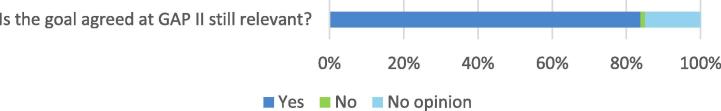
Relevance of the goal agreed at the GAP II consultation.

The survey asked whether this goal is still relevant. Of 74 responses received, the majority (84%) agreed that it was, and only one disagreed (Fig. 2), the rest giving no opinion.

### 5.2 Global progress made under the GAP

The survey asked respondents how they would rate global progress under each of the GAP’s three objectives (listed in the introduction), as well as progress across all three objectives as a whole.

**Fig. 3:**
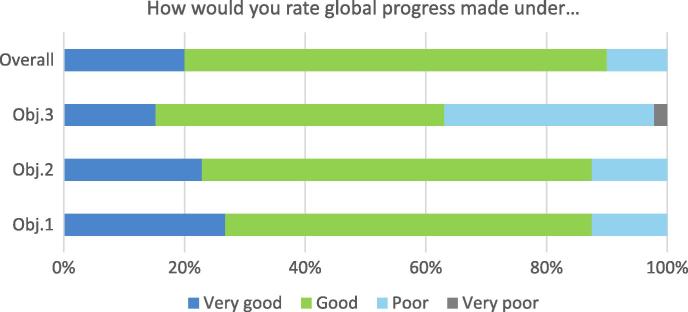
Global progress made under GAP objectives.

Respondents were least positive about progress under objective 3 (Fig. 3). A third (33%) considered global progress under objective 3 to have been poor, and one respondent thought it very poor - though several respondents felt that slow progress is to be expected given the nature of universal vaccine development challenges. Objectives 1 and 2 were considered to have made better progress: only an eighth of respondents for both objectives (12.5%) though progress was poor, and none thought it very poor. Respondents were similarly positive about overall progress across the three objectives.

With respect to field of work, respondents representing vaccine manufacturers were much more positive about progress towards objective 3. Conversely, their view is relatively negative about progress under objective 2.

## 6 Global progress made under GAP objectives 1, 2, and 3

Below is a summary of comments, both positive and negative, that respondents used to justify their rating of the progress made under the GAP:

Objective 1:

– *Distribution and use of seasonal vaccines has been increasing at the global level.*
– *Awareness of influenza’s contribution to the overall disease burden in many populations has been growing.*
– *Progress has been uneven - geographically and between sub-populations.*
– *Barriers to progress in further uptake persist in developing countries due to competitive health priorities, high costs, healthcare worker/public vaccination hesitancy, and low awareness.*

Objective 2:

– *Global production capacity has increased in terms of seasonal vaccines; the number of countries producing influenza vaccines; and strengthening of National Regulatory Authorities. Some seasonal influenza vaccines have received WHO pre-qualification status.*
– *Long-term sustainability is still questionable.*
– *Work remains to further streamline global activities and remove barriers to facilitate vaccine release and distribution.*

Objective 3:

– *More novel vaccines are now in early and late stages of development.*
– *Although high quality work has been carried out, there have not been any substantial advances in the influenza vaccine field in recent years (i.e. no “breakthrough discovery”).*

### 6.1 Main successes and shortcomings of GAP

Respondents were asked what they saw as the GAP’s main successes and shortcomings. Their responses are summarized in Table 1, with the most common themes listed first.

#### 6.1.1 Perception of factors influencing progress on GAP Objective 1

**Fig. 4:**
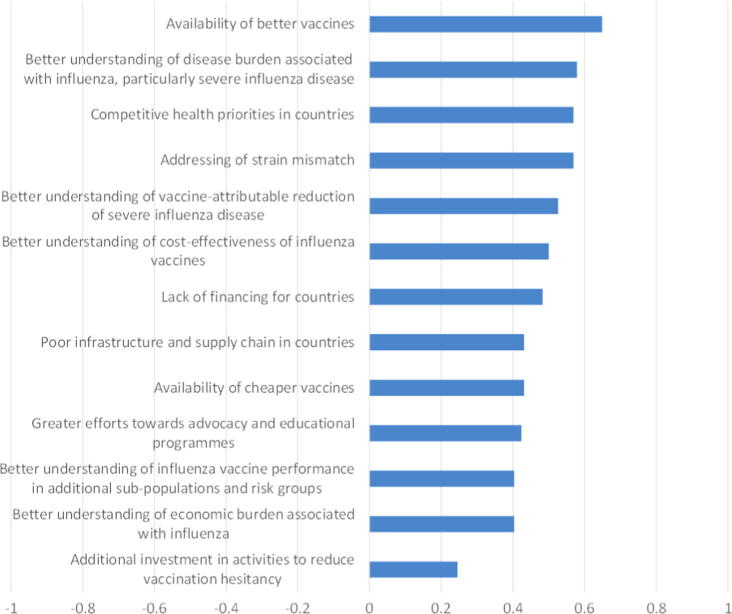
Importance of factors for facilitating introduction and uptake of seasonal influenza vaccines: average score (Very important = 1, Somewhat important = 0, Not important = −1).

Respondents were asked to rate the importance of different factors in terms of their role in facilitating the introduction and uptake of seasonal influence vaccines. Fig. 4 ranks the factors in order of importance based on the 58 responses received for this question.

All of the factors listed in Fig. 4 were considered at least somewhat important by the majority of respondents. The availability of better vaccines was considered the most important, while additional investment in activities to reduce vaccination hesitancy was considered least important.

**Fig. 5:**
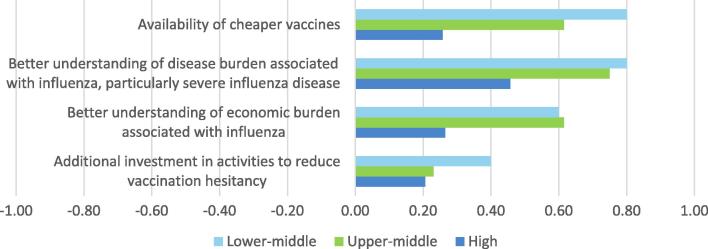
Importance of factors for facilitating the introduction and uptake of seasonal influenza vaccines - by WB income status: average score (Not important = −1, Somewhat important = 0, Very important = 1).

The summary provided by Fig. 4 masks some important differences of opinion between respondents from high and middle income countries. In particular, when results are broken down by income group it appears that, although cheaper vaccines are not considered to be particularly important by respondents from high income countries, the opposite picture prevails for upper and lower middle income countries (Fig. 5). Respondents from lower middle income countries also considered understanding of the disease and economic burdens associated with influenza, and investment in reducing vaccine hesitancy, to be more important for increasing seasonal uptake.

#### 6.1.2 Perception of factors influencing progress on GAP Objective 2

Survey participants were asked to rate the importance of several factors and their effect in ensuring that additional production capacity remains sustainable. The factor viewed as most important was a demonstrated value proposition for influenza vaccine programme investment in low and middle income countries (of 53 responses, 80% were “very important” and the rest “somewhat important”). Conversely, the factor deemed least important was the potential to export to other countries (49% of responses were “very important” and 6% did not think it was important at all).

**Fig. 6:**
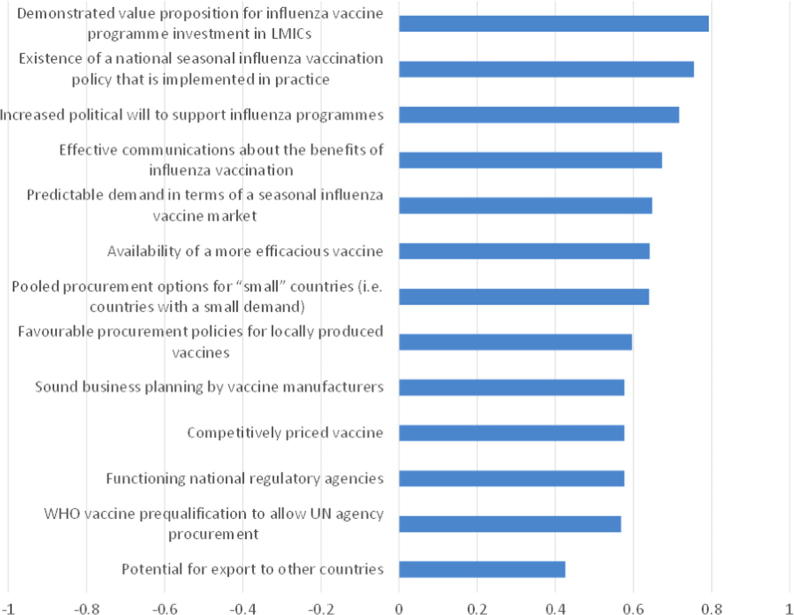
Importance of factors for sustaining existing vaccine production capacity: average score (Very important = 1, Somewhat important = 0, Not important = −1).

Again, the breakdown of responses in Fig. 6 conceals some variation due to country income levels. For example, high income countries ranked having functioning national regulatory agencies and competitively priced vaccines as the least important. Yet, in middle income countries these aspects were viewed as priorities. Favourable procurement policies for locally produced vaccines also divided opinion. Respondents from lower middle income countries ranked this factor as most important of all, but those from high income countries put it second lowest.

As expected, there was also variation in survey responses with regards to field of work. Respondents working in vaccine and pharmaceutical production viewed favourable procurement policies as more important than other respondents. Meanwhile, they viewed availability of a more efficacious vaccine as less important relative to other respondents.

#### 6.1.3 Perception of factors influencing progress on GAP Objective 3

The survey asked respondents to explain what they saw as the major challenges to research and development of better vaccines that elicit broader, longer lasting immune response, with respect to regulatory science; scientific knowledge; financial availability; intellectual property rights, and other factors. The 48 responses highlighted the following challenges:

##### 6.1.3.1 Regulatory science

• *Finding a regulatory pathway based on T-cell mediated correlates of protection, or more broadly, correlates beyond the haemagglutination-inhibition (HAI) assay method which may not be sufficient for testing new vaccine approaches.*
• *Clinical trial requirements may impose prohibitive time, information and resource costs given the returns available from producing influenza vaccines.*
• *Regulatory scrutiny in general presents a major challenge to timely production of effective vaccines.*
• *Requirements to license vaccines with regulatory agencies in multiple countries.*

##### 6.1.3.2 Scientific knowledge

• *Recombinant vaccines have relatively weak immunogenicity and require stronger adjuvants which are not yet licensed.*
• *The protective mechanism to influenza infection is not yet sufficiently well understood. The major challenge is finding a “universal” antigen for a changing influenza virus. The antigens required to develop a universal vaccine, or at least a vaccine with high cross protection against similar subtypes, are hard to identify.*

##### 6.1.3.3 Financing

• *In the absence of strong profit incentives, research and development of better influenza vaccines is dependent on government and donor commitments. Short-term, intermittent funding can also create challenges.*
• *The lack of a strong annual market for seasonal vaccines may diminish industry investment.*
• *Influenza faces strong competition from other global health vaccine challenges.*

##### 6.1.3.4 Intellectual property rights

• *Intellectual property rights are currently preventing full exploitation of synthetic generation and the reverse genetics of influenza viruses. Making reverse genetics accessible under more favourable financial terms would be welcome.*
• *Although intellectual property rights are important for encouraging innovation, they can also hamper technology transfer to low and middle-income country manufacturers.*

##### 6.1.3.5 Other factors

• *Maintaining commitments towards influenza research despite the frustration of recent years.*
• *Maintaining stakeholder collaboration.*

### 6.2 Cross-cutting issues

**Fig. 7:**
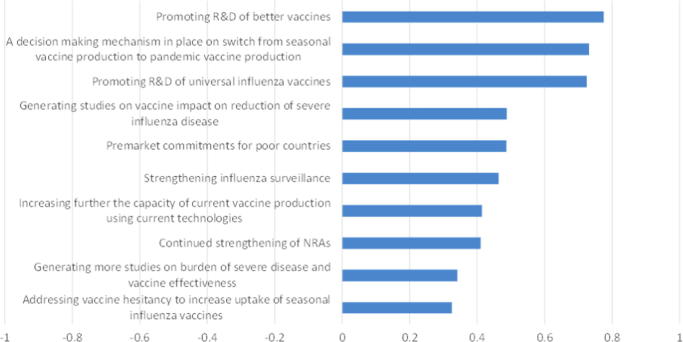
Priorities for ensuring timely vaccine availability in the event of a pandemic: average score (Very important = 1, Somewhat important = 0, Not important = −1).

Respondents were asked to rate the importance of steps to ensure timely availability of sufficient pandemic influenza vaccines. On average, three factors stood out as priorities, as shown in Fig. 7: promoting research and development of better vaccines; a decision-making mechanism for switching from seasonal to pandemic production; and promoting research and development of a universal influenza vaccine. Addressing vaccine hesitancy and generating more studies on disease burden and vaccine effectiveness were considered relatively unimportant in the event of a pandemic.

## 7 Limitations

The low participation of lower middle and low income country stakeholders is a limitation to the analysis, in particular the few African respondents, and the fact that the supply side (industry and research) was relatively well represented in relation to those representing the users of vaccine. Only 4 percent of respondents were from Africa, 12 percent from South East Asia, 17 percent from the Western Pacific and 7 percent from the Eastern Mediterranean, for a total of 40%. The European region and the region of the Americas counted for the majority of respondents (60%). Future surveys should place a stronger emphasis on recruiting participants from lower middle and low income countries.

The number of survey respondents is too low to permit informative statistical analysis of the results. However, this is not considered to be a major shortcoming since the results of this survey were used for descriptive, not inferential, purposes, rather than to demonstrate statistical significance. Therefore, the number of respondents is deemed sufficient for the purposes of gauging key GAP stakeholders’ opinions and contribute to the final GAP consultation in November.

Furthermore, the survey was only available in the English language, and in an-online, written format. Potential respondents from other languages or who do not have good access to the internet may have been deterred, though the majority of stakeholders targeted are expected to have had adequate internet connectivity and knowledge of English.

## 8 Conclusion

Full analysis of the perceptions obtained from this survey will feed into discussions at the GAP consultation in November 2016, where stakeholders will consider how funding and momentum for ongoing projects in countries might be affected by the GAP’s closure. Discussions will build on the findings that there is strong agreement on the relevance of the goal agreed at the GAP II consultation; and that relatively little global progress is perceived to have been made under GAP objective 3. Discussions will be informed by a better understanding of stakeholder priorities, the successes and challenges the GAP has faced, and barriers to future progress.

## Conflict of interest

The authors state they have no conflict of interest.

## Figures and Tables

**Table 1:** Perceived main successes and shortcomings of the GAP: summary table.

Successes	Shortcomings
• Capacity building in developing countries - both in terms of production and National Regulatory Authority strengthening • Raising international awareness in relation to the needs of the global community in the event of a pandemic • Collaborative alignment of influenza stakeholders in different countries and sectors • Increased global manufacturing capacity • Sharing evidence on the efficacy and effectiveness of seasonal vaccination in various populations • Assessing projected needs for global pandemic vaccine	• Insufficient dedicated resources to make a substantial and sustained impact • Limited progress in development of a broadly protective or universal vaccine • Failure to generate a major increase in demand for seasonal influenza vaccine • Vaccine production capacity and competencies are still in the hands of a few and not yet spread enough (i.e. to low and middle income countries) to face a pandemic • Product registration processes are still too slow
